# Nonlinear mid-infrared meta-membranes

**DOI:** 10.1515/nanoph-2024-0203

**Published:** 2024-07-24

**Authors:** Giovanni Sartorello, Joshua Bocanegra, David Knez, Daniil M. Lukin, Joshua Yang, Jelena Vučković, Dmitry A. Fishman, Gennady Shvets, Maxim R. Shcherbakov

**Affiliations:** School of Applied and Engineering Physics, 8788 Cornell University, Ithaca, NY 14850, USA; Department of Electrical Engineering and Computer Science, University of California, Irvine, CA 92697, USA; Department of Physics and Astronomy, University of California, Irvine, CA 92697, USA; Department of Chemistry, University of California, Irvine, CA 92697, USA; E. L. Ginzton Laboratory, Stanford University, Stanford, CA 94305, USA; Beckman Laser Institute and Medical Clinic, University of California, Irvine, CA 92697, USA; Department of Materials Science and Engineering, University of California, Irvine, CA 92697, USA

**Keywords:** metasurfaces, nonlinear optics, silicon carbide, mid-infrared

## Abstract

Nanophotonic structures have shown promising routes to controlling and enhancing nonlinear optical processes at the nanoscale. However, most nonlinear nanostructures require a handling substrate, reducing their application scope. Due to the underwhelming heat dissipation, it has been a challenge to evaluate the nonlinear optical properties of free-standing nanostructures. Here, we overcome this challenge by performing shot-controlled fifth harmonic generation (FHG) measurements on a SiC meta-membrane – a free-standing transmission metasurface with pronounced optical resonances in the mid-infrared (*λ*_res_ ≈ 4,000 nm). Back focal plane imaging of the FHG diffraction orders and rigorous finite-difference time-domain simulations reveal at least two orders of magnitude enhancement of the FHG from the meta-membrane, compared to the unstructured SiC film of the same thickness. Single-shot measurements of the meta-membrane with varying resonance positions reveal an unusual spectral behavior that we explain with Kerr-driven intensity-dependent resonance dynamics. This work paves the way for novel substrate-less nanophotonic architectures.

## 1 Introduction

Nonlinear nanophotonics [1] offers a route to optical signal processing [2], [3] and new light sources [4], [5] answering the growing demand for high-speed computing and data transfer. Various platforms for frequency mixing and all-optical switching have been established [1], [6]–[9]. Due to the weakness of nonlinear interactions, extended propagation lengths are required for reasonable frequency conversion efficiencies. This deficiency can be alleviated with photonic nanostructures [10–16] and metasurfaces [17–20], where the subwavelength thicknesses lift the phase-matching requirement via resonant excitation. Functionally, the metasurfaces’ compact footprint allowed for innovation in non-reciprocity and asymmetric image generation [21], [22], nonlinear holography and wavefront control [23]–[25], as well as beam steering [26]. In most cases, however, the metasurfaces require handling substrates for mechanical stability and integrity, significantly increasing their physical footprint and, as a result, limiting the scope of their potential applications.

Free-standing metasurfaces – or meta-membranes – represent an attractive alternative to metasurfaces on substrates. In meta-membranes, the active layer of the device is its only layer. The absence of the thick substrate can enable mechanical flexibility [27], eliminate substrate-superstrate asymmetry for mode engineering [28], [29], as well as facilitate bi-interfacial access to liquid environments for sensing and microfluidics [30], [31]. Exceptional linear-optical characteristics of free-standing metallic, dielectric and phonon-polaritonic metasurfaces, such as resonant reflectance, transmittance, absorbance, diffraction and polarization sensitivity, have been revealed [32–36]. However, poor thermal dissipation has made the studies of freestanding metasurfaces under strong laser excitation challenging, requiring a substrate for heat sinking [37].

In this paper, we report on the nonlinear optical responses of free-standing dielectric meta-membranes. A SiC-based holey metasurface exhibits collective resonances in the mid-infrared frequency range (*λ*_res_ ≈ 4,000 nm) and produces bright fifth harmonic generation (FHG; *λ*_FHG_ ≈ 800 nm) in the back focal plane (BFP) of the imaging system. By sending a precise number of laser pulses onto the sample, we measure that the single-pulse FHG is at least two orders of magnitude stronger than that from an unpatterned SiC membrane, as confirmed by rigorous nonlinear finite-difference time-domain (FDTD) simulations. We observe a quick pulse-to-pulse deterioration of the FHG intensity, with the sample damage occurring at different pulse numbers for different polarizations and producing different damage patterns. The FHG intensity is resonance-dependent with unusual FHG spectra shifts, which we explain with nonlinear Kerr-effect-driven resonance dynamics implemented in a coupled-mode theory and nonlinear FDTD. Our results open a promising perspective on the nonlinear free-standing metasurfaces and outline future directions of studies in nonlinear optics of substrate-less photonic devices.

## 2 Materials and methods

**Figure 1:**
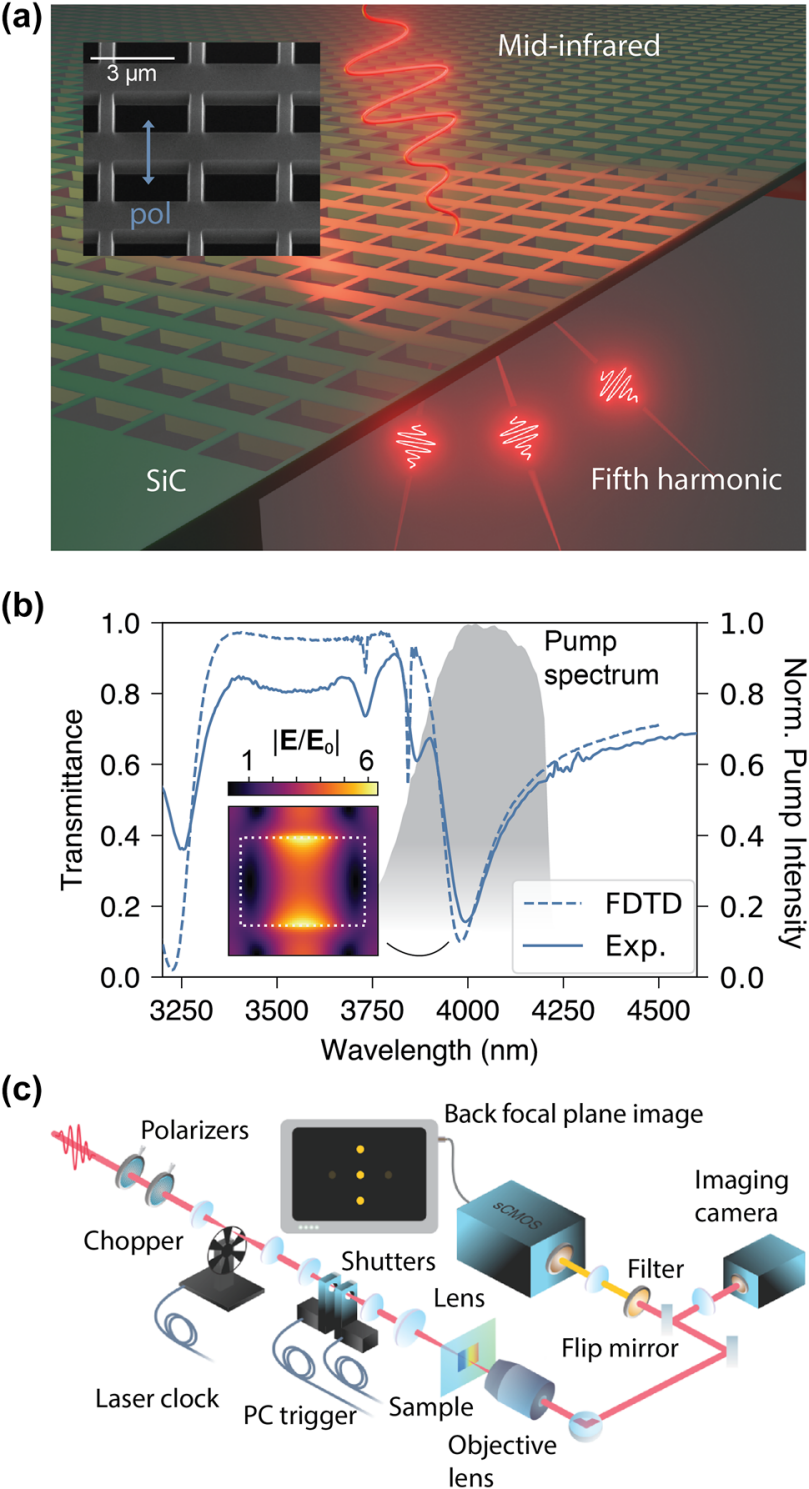
Sample and setup. (a) SEM micrograph (left) and schematic (right) of the sample. The arrays are fabricated out of a continuous SiC membrane of a varying thickness. (b) An experimental mid-IR transmittance spectrum of the sample for the design polarization (solid line) and the corresponding FDTD calculation (dashed line). The spectrum of the pump laser is superimposed (gray shaded area). The calculated local field distribution at a wavelength of 4,000 nm in the middle section of the meta-membrane is shown to provide a substantial field enhancement at the pump wavelength. (c) Schematic of the experimental FHG BFP imaging setup.

The sample of a meta-membrane was fabricated from a SiC free-standing film starting with a monocrystalline 4H-SiC on Insulator (SiCOI) substrate fabricated as described in Ref. [38]. The substrate for the SiCOI film is 500 μm thick silicon. The metasurface is defined via electron-beam lithography and etched using an aluminum hardmask as described in Ref. [39]. To remove the substrate underneath the metasurfaces, we pattern a chromium hard-mask on the backside of the sample and etch 400 μm deep square hole via a high-selectivity reactive ion deep silicon etch (PlasmaTherm Versaline DSE). The sample is transferred to a XeF_2_ gas etch system (Xactics e-1) to gently etch away the remaining 100 μm of silicon beneath the metasurface. Finally, the remaining silicon dioxide layer beneath the SiC is removed via a vapor HF etch (SPTS uEtch). Mechanical rigidity of the SiC membrane is ensured via a moderate tensile stress (170 MPa) of the membrane [40]. The overall membrane size is 3.5 × 3.5 mm^2^, supported on all sides by the residual of the Si wafer used during its fabrication for mechanical stability. Most of the free-standing area was patterned with identical rectangular holes with *w*_*x*_ = 2.3 μm and *w*_*y*_ = 1.6 μm, arranged in a square array with a period of *p*_*x*_ = *p*_*y*_ = *p* = 2.7 μm. The meta-membrane was designed to resonate in the mid-infrared around *λ*_res_ ≈ 3.7 −4.3 μm. A typical SEM image of the membrane and its schematic is given in Figure 1(a). The natural thickness variation of the SiC film between 700 nm at the center and 450 nm at the edge allowed varying the resonance position across the desired range. The unpatterned parts of the membrane were used for control measurements.

Fourier-transform infrared spectroscopy (FTIR) was performed using a Bruker Vertex 80v spectrometer with an external home-built arm described elsewhere [41]. The minimum spot size is less than 200 μm in diameter, allowing mapping the metasurface frequencies across the sample and for two select linear polarizations (horizontal and vertical).

FHG experiments were performed in a shot-controlled setting using a mid-infrared system based on a Ti:sapphire amplifier, optical parametric amplifiers, and difference-frequency generators; see Figure 1(c) for the setup schematic. We used a multimodal laser system consisting of both high repetition rate oscillator (MaiTai HP, 3 W, <80 fs) and low repetition rate amplifier (Spitfire Ace, 1 kHz, 100 fs, >7 W). The fundamental beam was split into four parts. Two of the resulting beams were used to pump two optical parametric amplifiers (OPA, Light Conversion TOPAS Prime™) for generating broadband optical pulses, tunable over the extended range from 280 to 2,700 nm. In combination with difference frequency mixing, the accessible range is extended up to 20 μm (>10 μJ at 15 μm). To avoid water absorption for wavelengths above 4,000 nm, the design wavelength of *λ*_pump_ = 4,000 nm was chosen. The temporal pulse width after each OPA was controlled by a prism compressor to compensate for the setup’s dispersion, and for our experiments, it was characterized to be close to Fourier-transform-limited (*τ* ≈ 200 fs).

FHG was measured in transmission, with focusing of the fundamental beam by a singlet plano-convex *f* = 100 mm lens (focal spot diameter ≈120 μm), collection of the harmonics by a Leica N Plan L 50×, NA = 0.50 objective, and detection by either of two cameras. The main camera (Teledyne Prime BSI CMOS) is set up for BFP imaging and used to detect the FHG passing through a bandpass filter (Thorlabs FBH800-40). The second camera (Andor Clara E CCD) is accessed by flipping a mirror for sample focusing and navigation. The setup is capable of repeatable single-shot measurements by using an optical chopper with a 10:1 wheel sequenced with two synchronized shutters (Thorlabs SH05R) set to open and close inversely. The beam exposure time is, therefore, below the 10 ms gate, equivalent to a single-pulse irradiation. Here, the system was set up to provide a controllable number of femtosecond pulses (*N* = 1 to 1,000) at up to 6 μJ per pulse to the sample, with irradiances at the sample plane between 100 and 900 GW/cm^2^.

Numerical simulations were done using Ansys Lumerical FDTD on a 12-core Ryzen 5945wx system with 256 Gb of memory. Local field profiles were created with a unit cell consisting of a SiC with *p*_*x*_ = *p*_*y*_ = 2.73 μm and a varying thickness *h* = 0.45 −0.7 μm, with an etched rectangle of *w*_*x*_ = 2.35 μm, *w*_*y*_ = 1.64 μm. Importantly, the transmittance spectra only match the experimental ones well if a finite mid-IR beam size is considered. For that, we copied the unit cell in a 100 × 100 array and utilized a Gaussian beam with a 80-μm waist in the sample plane. The material dispersion was included separately in the fundamental, third harmonic, and fifth harmonic regions; see Supplementary Materials, Figure S4.

For the FHG FDTD calculations, the sample was illuminated with a 100-fs femtosecond laser pulse centered around *λ*_pump_ = 4,000 nm, and SiC was modeled with $\chi^{\left(1\right)}=5.5$ and $\chi^{\left(3\right)}=2\cdot 10^{-20}$ m^2^/V^2^, providing a path to the cascaded FHG through the $\chi^{\left(3\right)}\left(3\omega +\omega +\omega \right)$ mixing of the third harmonic generation and the pump radiation. The value of $\chi^{\left(3\right)}$ was selected to match that experimentally determined [42] $\chi_{\exp}^{\left(3\right)}\approx 2\cdot 10^{-20}$ m^2^/V^2^, albeit in a different spectral range within the mid-infrared. While the direct $\chi^{\left(5\right)}$ FHG process is expected in SiC, we do not consider it in our calculations, as the cascaded effective $\chi_{\text{eff}}^{\left(5\right)}\approx \chi^{\left(3\right)}\left(3\omega =\omega +\omega +\omega \right)\chi^{\left(3\right)}\left(5\omega =3\omega +\omega +\omega \right)$ is known to be responsible for a large amount of FHG in many materials [43]–[45] and is sufficient to qualitatively explain most of the observed experimental data as evidenced below. The peak electric field strength is set at *E* = 2 × 10^9^ V/m, corresponding to a peak intensity of ≈500 GW/cm^2^. The far-field diffraction patterns were computed at the fifth harmonic frequency through the standard near-field-far-field projection and plotted at *λ*_FHG_ = 800 nm. Unless otherwise stated, all FDTD simulations run for 4,000 fs and have a *z*-mesh step size of 6.25 nm in the area of the sample.

## 3 Results

The transmittance of the patterned membrane depends on location, with a typical example shown in Figure 1(b). Linear polarization is used for all measurements. For the design polarization of the sample (V, along the short sides of the rectangular holes), there are relatively sharp resonances with the Q-factor in the range of 22–31 across the membrane in the desired spectral region at *λ*_pump_ ≈ 4,000 nm. Changes in the thickness of the membrane shift the resonance, and similar measurements performed in other positions on the sample give similar spectra and resonance wavelengths in the *λ*_res_ = 3,900 − 4,100 nm range (Supplementary Figure S2). The laser spectrum, shown with the gray shaded area in Figure 1(b), is broader than most of the observed resonances and covers the entire resonance region for the whole sample. The calculated local field mode profile taken in the middle cross-section of the meta-membrane in the inset of Figure 1(b) shows up to 6-fold electric field increase in when excited at the resonance wavelength.

**Figure 2:**
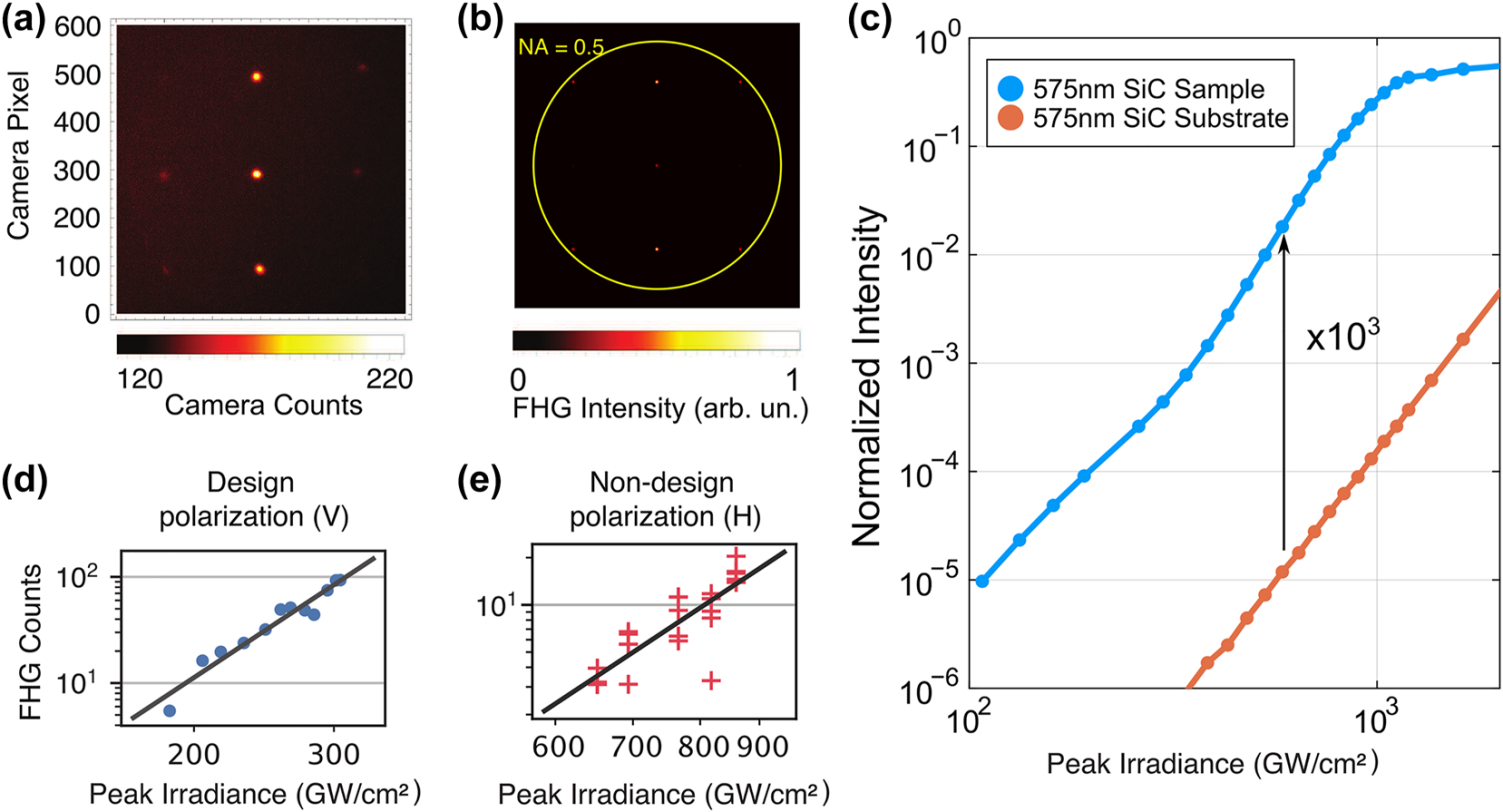
Single-shot FHG from the free-standing SiC meta-membrane. (a) BFP of the FHG emission from the metasurface for the vertical (V, design) polarization. (b) Simulated BFP image of FHG from a resonantly pumped metasurface (pump polarization V). The yellow circle denotes the numerical aperture of the collection system used in the experiment. (c) Calculated zeroth-order FHG intensity as a function of pump irradiance. An average enhancement of about 10^3^ is observed before saturation starts at an irradiance of around 1 TW/cm^2^. (d, e) Log-log-plotted intensity-dependent FHG for V and H polarizations, respectively, showing the data points and exponential fits *y* = *ax* ^*b*^. The fitted exponents are *b*_V_ = 5.0 ± 0.4 and *b*_H_ = 4.9 ± 0.7, respectively, closely matching the expected power relationship $I_{\text{FHG}}\propto I_{\text{pump}}^{5}$ in the perturbative regime.

**Figure 3:**
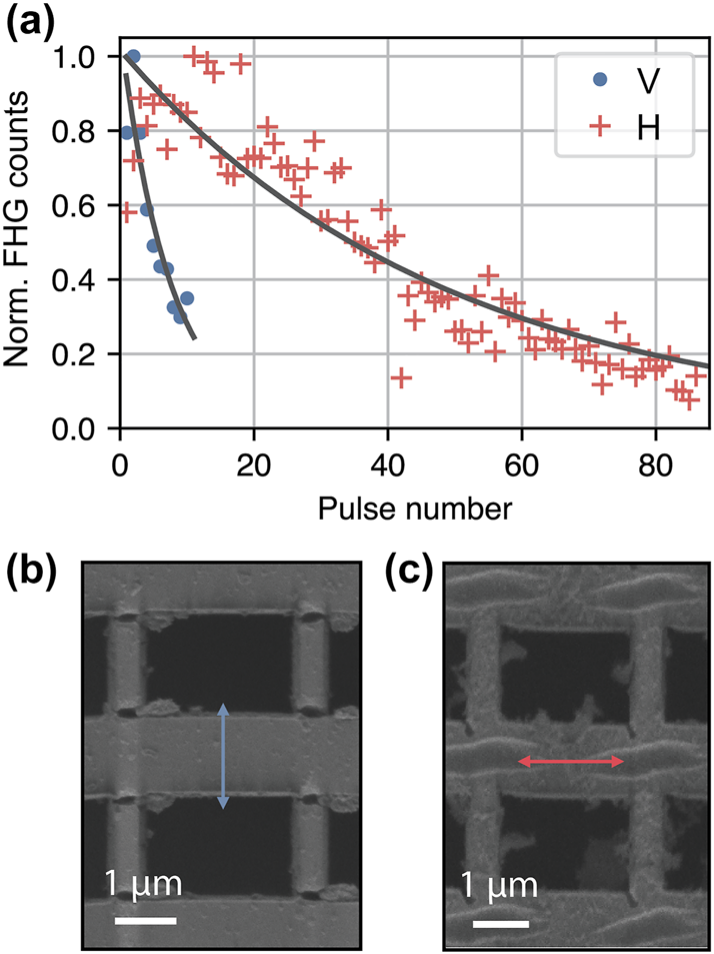
Shot-by-shot performance and damage. (a) FHG as a function of the number of consecutive pulses sent to the sample for vertically (blue circles) and horizontally (red crosses) polarized pump. Fit curves are exponentials with decay constants *N*_V_ = 7 pulses (*R* ^2^ = 0.98) and *N*_H_ = 49 pulses (*R* ^2^ = 0.96) for vertically and horizontally polarized pump, respectively. The decay is much faster for the design polarization (V) due to the mechanical structure of the metasurface unit cell. (b) The result of the metasurface irradiation by 100 V-polarized pulses. (c) The result of the metasurface irradiation of 100 H-polarized pulses. The white arrows indicate typical multi-shot damage propagating perpendicular to the polarization of the pump [46].

A typical FHG BFP image in Figure 2(a) shows several orders of diffraction for the fifth harmonic wavelength *λ*_FHG_ ≈ 800 nm, as selected with a bandpass filter in front of the BFP camera. The numerical aperture of the objective NA = 0.5 is sufficient to collect orders up to (0, ±1) and $\left(\pm 1,0\right)$ , while orders (±1, ±1) were on the edge of the reception angle of the objective lens. For analysis purposes, in this work, only order (0,0) is considered. It should be noted that the presence of the diffraction orders and the absence of incoherent emission filling the whole objective aperture signifies the absence of parasitic emission, such as multiphoton photoluminescence. The zeroth order is fitted in each BFP image with a 2D Gaussian, and the peak number of FHG counts is derived from each measurement. Figure 2(b) shows a BFP image calculated using nonlinear FDTD and measuring the far-field distribution at the fifth harmonic frequency. The calculations realistically capture the main diffraction features of the FHG observed experimentally. Additionally, nonlinear FDTD captures the power scaling of the FHG in Figure 2(c) for both the SiC substrate (orange), which demonstrates an expected fifth-power scaling, and the sample (blue), which adheres to the fifth-power scaling until saturation begins at an irradiance of approximately 1 TW/cm^2^. The nature of this saturation will be discussed in the Discussion section of the paper. The FHG nature of the experimentally detected signal is additionally proven with single-shot measurements performed while increasing the pump intensity. Fitted in the log–log scale in Figure 3(d) and (e) with *y* = *ax* ^*b*^, the resulting slopes are *b*_V_ = 5.0 ± 0.4 (*R* ^2^ = 0.98) and *b*_H_ = 4.9 ± 0.7 (*R* ^2^ = 0.95), signifying the perturbative regime of harmonic generation. Note that, because of the difference in the Q-factor and the local field enhancement, the overall FHG counts are significantly higher in the case of V ( ≈100 counts at 300 GW/cm^2^) than in the case of H ( ≈10 counts at 800 GW/cm^2^).

The local field enhancement provided by the resonance at the pump wavelength tremendously boosts the FHG efficiency. In fact, no single-shot FHG was observed for any pump intensity used in the experiment from an unstructured membrane, which is a reasonable comparison target as it carries no resonant response, including Fabry–Perot modes, being only several hundred nanometers in thickness. To provide a lower boundary to the potential FHG enhancement, we compare the highest single-shot FHG count obtained in the experiment (94; see Figure 2(d)) to the typical noise of FHG count determination; the latter is derived from the fit to a Gaussian of the FHG diffraction orders and is around 0.5 counts, providing at least two orders of magnitude in FHG enhancement. The true enhancement by the metasurface can be appreciated with FDTD calculations, as shown in Figure 2(c). Here, the ratio of the zeroth order FHG emitted by the meta-membrane and the unstructured substrate is 10^3^, substantiating our claim of at least two orders of magnitude in FHG enhancement by the meta-membrane.

We observed that several shots in the same position would permanently damage the metasurface, as long-term laser damage tends to accumulate in resonant nanostructures [46]. To characterize the accumulated effect of sample damage on the FHG, we repeatedly performed single-shot measurements at the same position on the sample for both polarizations. Figure 3(a) shows the FHG as a function of the shot number for V and H polarizations. Shots are released manually with sufficient time between each shot (>1 s) that cumulative thermal effects can be neglected. With the design polarization (blue dots) decay is much faster than with the other one (red crosses). Exponential fits (solid lines) give decay constants of 7 and 49 pulses for the two polarizations, respectively.

The insight in the disparate behavior of FHG on the shot number can be acquired through microscopy images shown in Figure 3(b) and (c) for the V and H polarizations, respectively. Consistent with our previous studies [46], the multi-pulse damage occurs in the form of a nanotrench propagating perpendicular to the polarization of the beam. The design polarization seeds the trench across the narrower beam of the meta-membrane, which is easier to break than that of the non-design polarization. These observations pinpoint an important mechanical aspect of light–matter interactions at the nanoscale that requires to be co-designed along with the purely electromagnetic aspects of light–matter interactions.

**Figure 4:**
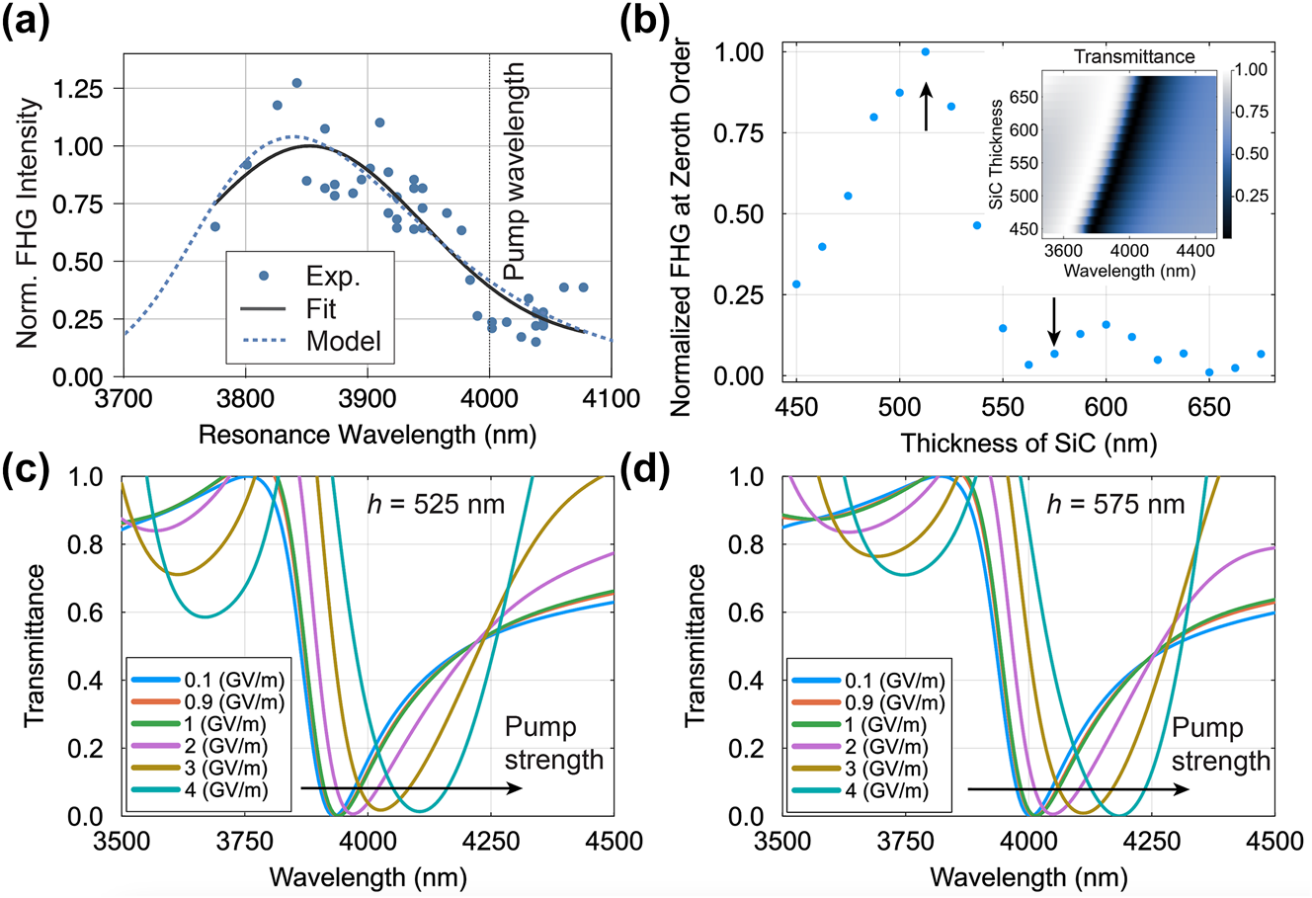
Kerr-assisted FHG. (a) Experimental single-shot FHG as a function of the meta-membrane’s resonance (blue dots). The resonance wavelength is derived from the sample thickness map by using FTIR microscopy and numerical simulations. Data are fitted with a Gaussian (solid line), yielding peak FHG generation at 3,850 nm. The laser carrier wavelength is shown with a vertical dashed line, indicating an unusual behavior where the maximum FHG signal occurs when *λ*_res_ ≠ *λ*_pump_. A Kerr-nonlinearity-driven model describes this behavior, producing the dotted curve; see text for details. (b) Zeroth-order FHG as a function of SiC membrane thickness at a field strength of 2 GV/m. FHG peaks at a thickness of *h* = 525 nm, at which the low-field resonance is blue-shifted. The inset shows the linear transmittance spectrum of the meta-membrane calculated at different thicknesses. Arrows indicate the cases presented in panels (c) and (d). (c) The transmittance of the meta-membrane at a thickness of *h* = 525 nm as a function of the pump field strength. The resonance is red-shifted and overlaps better with the pump, generating a high level of FHG. (d) Same for *h* = 575 nm, where the resonance is departing away from the pump at high field values. Larger-than-unity transmission in panels (c) and (d) indicates frequency mixing in the pump beams under strong-field excitation.

To study the resonance-dependent behavior of FHG, sequences of single-shot measurements, each on a fresh spot of the sample, were performed going across it in places with differing thicknesses for the design polarization (V). The position of each shot was recorded, and its thickness was derived by using a thickness map of the membrane obtained before it was patterned; see Supplementary Figure S3 for the sample map, including shot positions and thickness. From the thickness, the resonance wavelength of the patterned membrane at each point can be reconstructed by comparison with numerical simulations. This analysis allows the FHG counts to be plotted as a function of the resonance wavelength. In Figure 4(a), the FHG counts for the single-shot measurements are shown for resonances with different wavelengths across the sample. The data was fitted to a Gaussian (solid line), disclosing a peak around 3,850 nm. The difference in the peak position for the FHG as a function of the resonance wavelength is attributed to the dynamic resonance red shift; see Discussion.

## 4 Discussion

Figure 4(a) shows an unusual FHG behavior – the highest FHG signal is produced by the parts of the meta-membrane with resonance wavelengths that are shorter than the pulse wavelength *λ*_res_ < *λ*_pump_. We observe that counts decrease as the resonance wavelength of a given position on the sample approaches the peak laser wavelength 4,000 nm. This implies that the actual resonance of the sample, when under the effect of a laser pulse, is redshifted. For example, a region with 3,850 nm “static” resonance will have its refractive index modified by the pulse, and its “dynamic” resonance will be more in the range of 4,000 nm, the carrier wavelength of the pump.

The apparent dynamic redshift of the resonance and the explanation as to why the peak FHG is observed for the parts of the sample with a blue-shifted resonance can be explained in the context of the Kerr-type frequency shift induced by the strong laser pulses. We employ a coupled-mode theory (CMT) model [47]–[49] with an intensity-dependent resonance position:

$$\dot{a}\left(t\right)+i\left(\omega -\kappa {\left|a\left(t\right)\right|}^{2}\right)a\left(t\right)+\gamma a\left(t\right)=\sqrt{\gamma }E\left(t\right),$$

where $a\left(t\right)$ is the mode complex amplitude, *ω* is the stationary mode frequency swept from $\omega_{\text{res}}^{\left(1\right)}=460$ rad/ps $\left(\lambda_{\text{res}}^{\left(1\right)}=\frac{2\pi c}{\omega }=4100\;\text{nm}\right)$ to $\omega_{\text{res}}^{\left(2\right)}=509$ rad/ps ( $\lambda_{\text{res}}^{\left(2\right)}=3700$ nm) to, *γ* = 27 rad/ps is the radiative damping, and $E\left(t\right)=E_{0}\exp\left[-t^{2}/\tau^{2}-\text{i}\omega_{0}t\right]$ is a Gaussian-envelope pump-field with *τ* = 100 fs and *ω*_0_ = 470 rad/ps (*λ*_pump_ = 4,000 nm). The fifth harmonic intensity is calculated in the arbitrary units as $I_{\mathrm{FHG}}\propto {\left|a^{5}\left(t\right)\right|}^{2}$ . The choice of coefficients *κ* and *E*_0_ is done to fit the resulting $I_{\mathrm{FHG}}\left(\lambda_{\text{res}}\right)$ to the experimental data; see Supplementary Materials for the code used to calculate $I_{\mathrm{FHG}}\left(\lambda_{\text{res}}\right)$ . The resulting modeled dependence is shown in Figure 4(a) with a dashed curve, showing agreement with the experimental data. Importantly, here, the required redshift of the resonance $\Delta \omega =\kappa {\left|a_{\max}\left(t\right)\right|}^{2}=7$ rad/ps requires a large refractive index change of roughly $\Delta n=\frac{n\Delta \omega }{\omega }\approx 0.05$ . However, given the known value [42] of SiC’s Kerr nonlinear index *n*_2_ ≈ 5 × 10^−6^ cm^2^/GW, and given the typical irradiances in our experiment *I*_pump_ ≈ 300 GW/cm^2^, and the quality factors that tend to enhance the local electromagnetic fields *Q* ≈ 25, the Kerr-induced nonlinearity enhanced by the resonant fields can reach Δ*n*_Kerr_ ≈ *n*_2_ *QI*_pump_ ≈ 0.04. Being the same order of magnitude, this nonlinear refractive index shift can reasonably explain the observed resonance redshifts and qualitatively substantiate the unusual dependence of FHG on the meta-membrane’s resonance position.

We further validate the simplistic CMT approach by rigorous calculations using nonlinear FDTD. Figure 4(b) shows FHG intensity at the zeroth diffraction order as a function of the SiC thickness for a pump field strength of 2 GV/m. The inset of Figure 4(b) shows the linear transmittance as a function of thickness, mapping the resonance position to the SiC thickness. As evidenced by these plots, in agreement with the experimental and CMT results, the thickness at which the FHG signal is the highest (*h* = 525 nm) is the one that initially shows a blue-shifted resonance. Conversely, the resonant meta-membrane (*h* = 575 nm) demonstrates a low FHG output. This behavior can be understood in the context of the Kerr-induced resonance dynamics. Figure 4(c) and (d) show the meta-membrane transmittance for different pump field strengths for off- (*h* = 525 nm) and on-resonance (*h* = 575 nm) excitation. While the resonance sweeps around the pump wavelength in Figure 4(c), enabling its efficient coupling to the meta-membrane, Figure 4(d) shows on-resonance excitation that gradually moves the resonance away from the pump spectrum, significantly lowering the FHG output. The difference in the resonance behavior unveiled by nonlinear FDTD elucidates the physics behind the experimental data and provides a promising perspective on predicting the nonlinear optical properties of meta-membranes under ultrastrong laser fields.

## 5 Conclusion

In conclusion, we have reported on the nonlinear optical response of resonant meta-membranes under shot-controlled femtosecond pulse irradiation. The SiC meta-membranes designed to host resonances in the mid-infrared have generated a pronounced fifth harmonic observed experimentally in the back focal plane as several diffraction orders. The harmonic intensity strongly depends on the resonant behavior of the meta-membrane, showing at least two orders of magnitude enhancement compared to an unstructured membrane on the same thickness in both experiments and simulations. The mechanical structure of the meta-membrane plays a crucial role in its pulse-to-pulse stability, where the laser-induced damage induced drastically different responses for two orthogonal polarizations. The harmonic intensity as a function of the resonance position reveals an unusual behavior explained by a Kerr-nonlinearity-driven dynamic resonance shifts, as well as rigorous nonlinear FDTD simulations. These results reveal new opportunities for substrate-less photonic devices in strong electromagnetic fields, with the potential applications in mid-infrared photonics, beam forming, novel light sources on a chip and high-power laser systems and components.

## Supplementary Material

Supplementary Material Details

Supplementary Material Details
