# Supplementary material for: Nonlinear mid-infrared meta-membranes
Source: Nanophotonics. 2024 Jul 24;13(18):3395–402. doi: 10.1515/nanoph-2024-0203 (PMC11501238; doi:10.1515/nanoph-2024-0203)
Supplement: Supplementary file 2 — Supplementary Material Details [file j_nanoph-2024-0203_suppl_002.pdf]

Nonlinear Mid-infrared Meta-membranes.

Supplementary Information

Giovanni Sartorello,[1] Joshua Bocanegra,[2,3] David Knez,[4] Daniil M. Lukin,[5] Jelena Vučković,[5] Dmitry Fishman,[4] Gennady Shvets[1], Maxim R. Shcherbakov,[2,6,*]

[1]School of Applied and Engineering Physics, Cornell University, Ithaca, NY 14850, USA

[2]Department of Electrical Engineering and Computer Science, University of California, Irvine, CA 92697, USA

[3]Department of Physics, University of California, Irvine, CA 92697, USA

[4]Department of Chemistry, University of California, Irvine, CA 92697, USA

[5]E. L. Ginzton Laboratory, Stanford University, Stanford, CA 94305, USA

[6]Beckman Laser Institute and Medical Clinic, University of California, Irvine, CA 92697, USA

*Corresponding author info:

2200 Engineering Hall, Irvine, CA 92697, USA maxim.shcherbakov@uci.edu

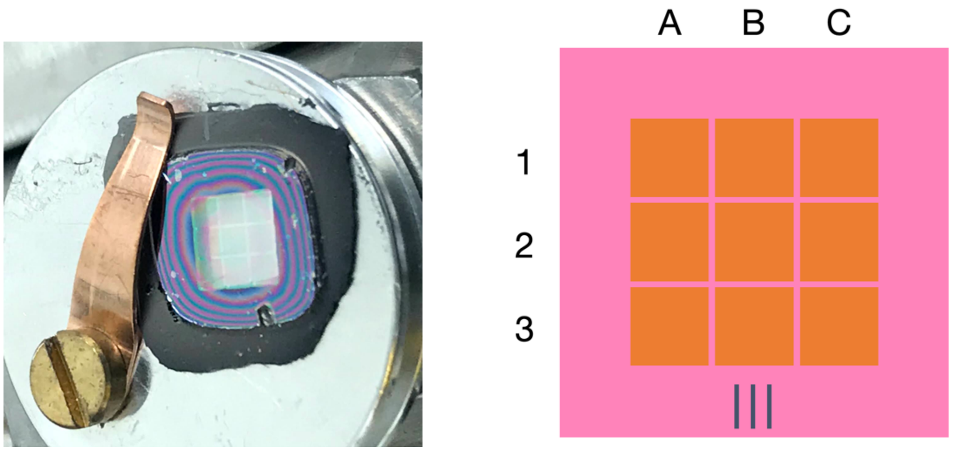

Supplementary Figure 1. (Left) A photograph of the meta-membrane sample before the FHG measurements. The middle of the sample shows 3x3 squares approximately 1x1 mm^2 each imprinted in the free-standing SiC film. The colorful rings on the surface of the sample indicates an uneven SiC film thickness, varying from 450 nm to 700 nm across the sample. (Right) The sample area designations.

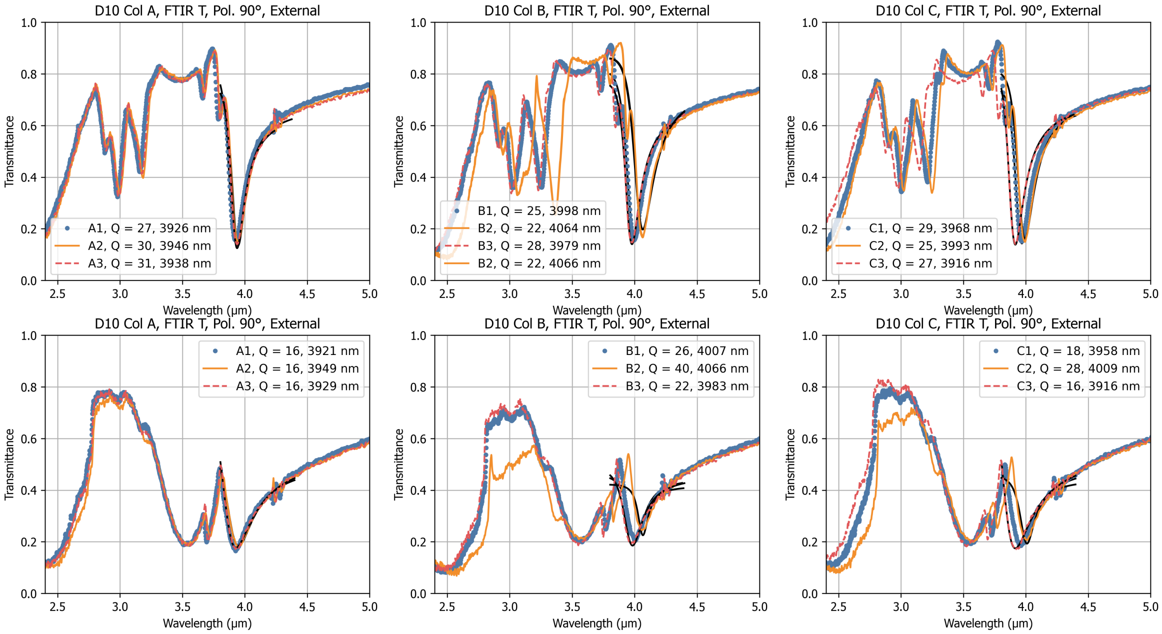

Supplementary Figure 2. FTIR spectra for the two polarizations, vertical (top row) and horizontal (bottom row). Each panel shows three different rows (1-3) within the same column (A, B or C). The resonances around 4000 nm are fitted to a Fano resonance profile, and the quality factor is calculated for each resonance (see plot legends).

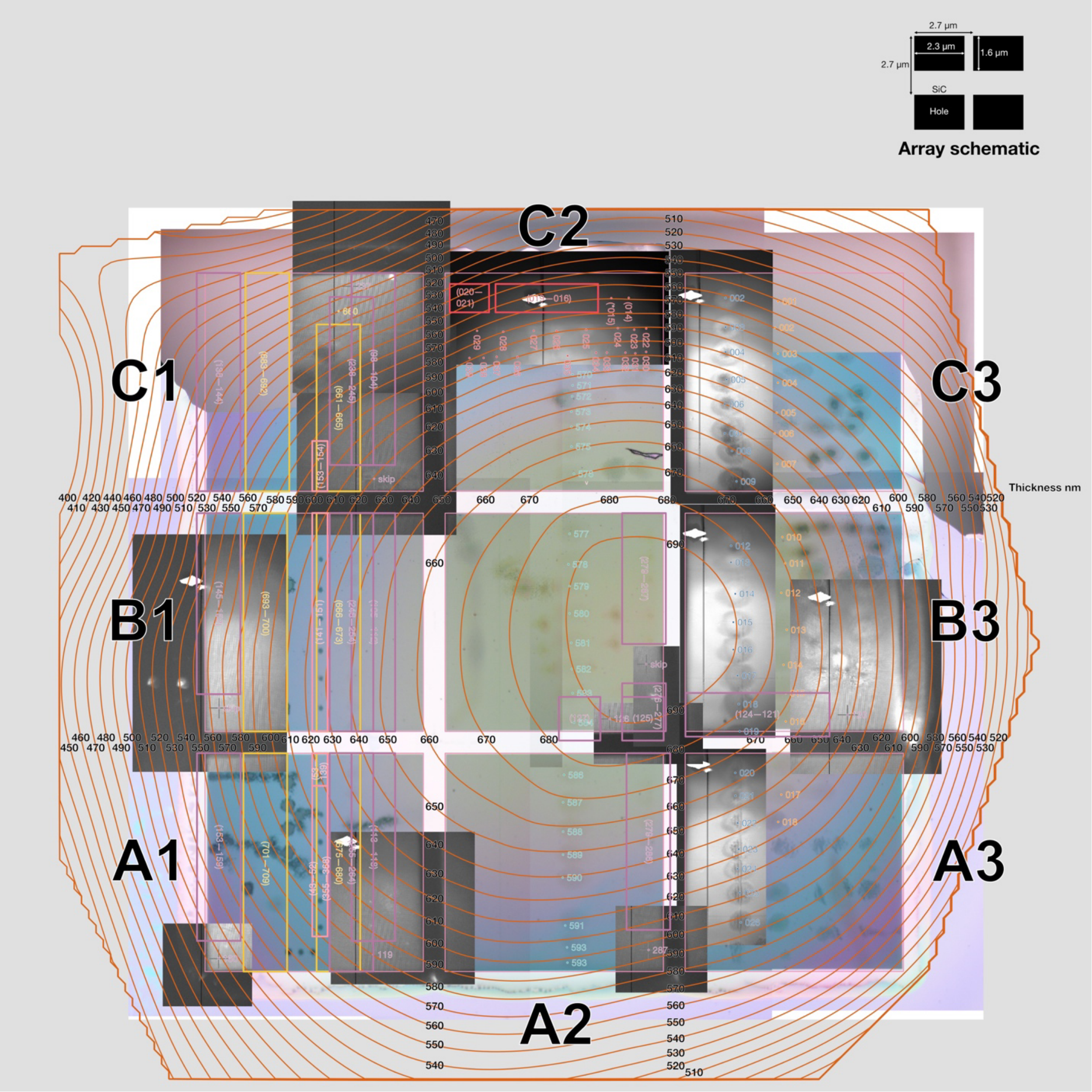

Supplementary Figure 3. Post-experiment optical and SEM composite image of the sample with relief map superimposed. Arrays are marked in black (except B2, at the center). Experiments are numbered and marked in color. More than 700 unique numbered experiments were performed on the sample. For each experiment, the FHG BFP image, pump intensity, number of pulses, their polarization, and sample thickness were recorded. The orange contour map shows the thickness distribution of the SiC membrane before the patterning.

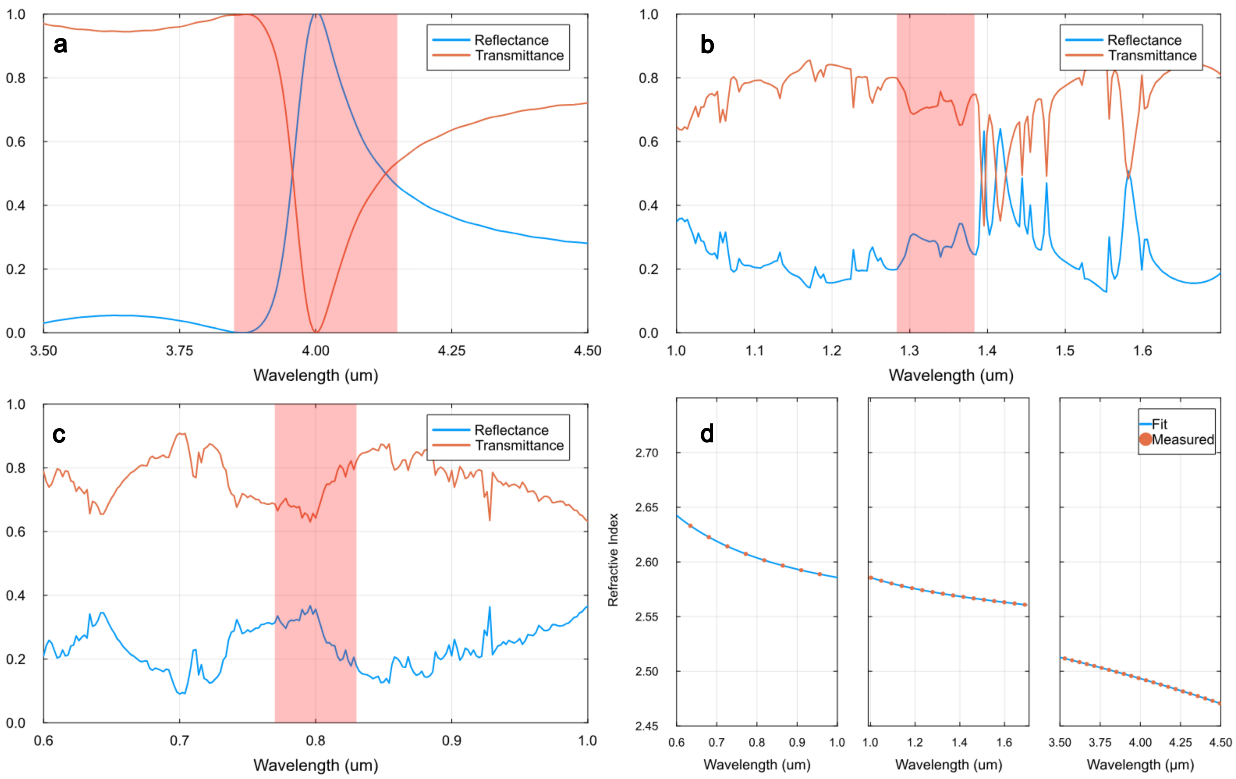

Supplementary Figure 4. **a**, Transmittance and reflectance of the meta-membrane calculated in the fundamental frequency range. The red rectangle indicates the approximate bandwidth of the pump pulse. **b**, Transmittance and reflectance of the meta-membrane calculated in the third harmonic frequency range. The red rectangle indicates the approximate bandwidth of the third harmonic radiation. **c**, Transmittance and reflectance of the meta-membrane calculated in the fifth harmonic frequency range. The red rectangle indicates the approximate bandwidth of the fifth harmonic radiation. **d**, The refractive index values used for calculations in a, b, and c. [S. Wang, M. Zhan, G. Wang, H. Xuan, W. Zhang, C. Liu, C. Xu, Y. Liu, Z. Wei, X. Chen. 4H-SiC: A new nonlinear material for midinfrared lasers, *Laser Photonics Rev.* **7**, 831–838 (2013)]

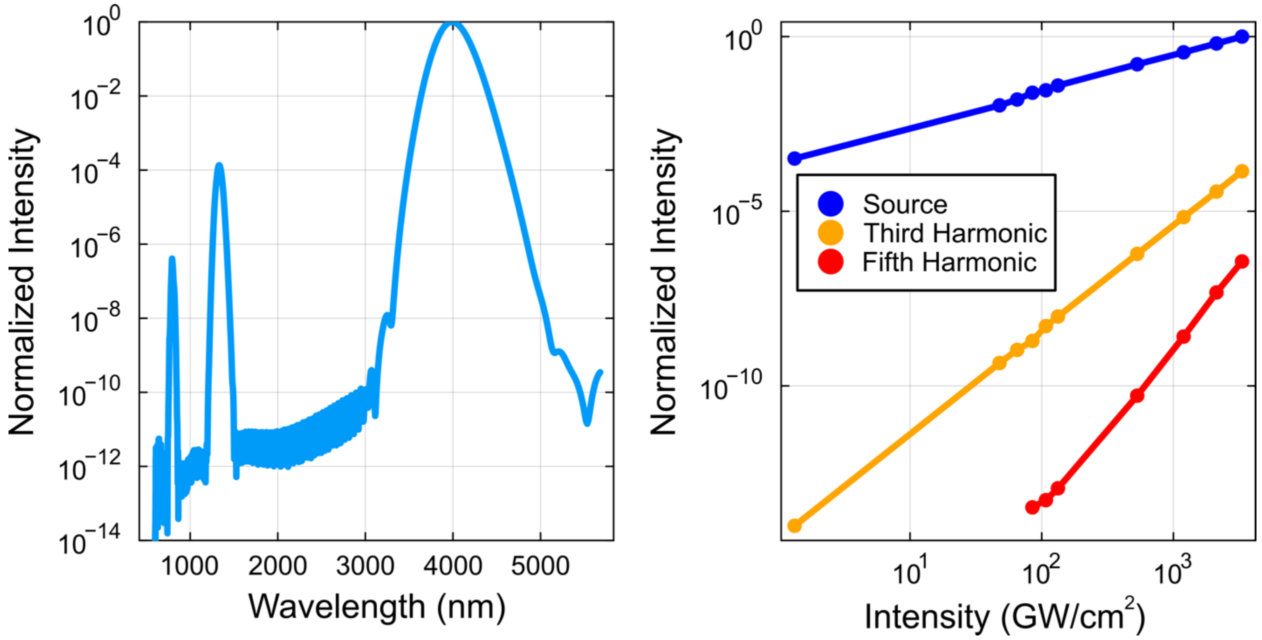

Supplementary Figure 5. (left) Fourier transform of the transmitted signal from an unstructured SiC film excited at $\approx$ 3 TW/cm^2 revealing the fundamental beam, the third and the fifth harmonics. (right) Intensities of the fundamental (source, blue), third (orange) and fifth harmonics (red) as functions of the fundamental beam intensity, showing linear, cubic and fifth-power growth and signifying a perturbative regime of harmonics generation.
